# Extension of a Coronary Intramural Hematoma as a Complication of Early Percutaneous Coronary Intervention after Thrombolytic Therapy

**DOI:** 10.1155/2013/218389

**Published:** 2013-06-09

**Authors:** Mohamed El-Mawardy, Mohamed Abdel-Wahab, Gert Richardt

**Affiliations:** Heart Center, Segeberger Kliniken GmbH, Academic Teaching Hospital of the Universities of Kiel and Hamburg, Bad Segeberg, Germany

## Abstract

The optimal treatment approach for coronary intramural hematomas has not been well defined, and discussion is limited to scarce data. In addition, the impact of prior thrombolytic therapy in the setting of coronary artery dissections with possible development and/or extension of an intramural hematoma is not well understood. We describe a case of iatrogenic periprocedural dissection of the left anterior descending artery (LAD) with development of an intramural hematoma and the extension of this hematoma to the left main (LM) and left circumflex (LCX) arteries in a middle-aged female, where prior recent thrombolytic therapy may have played a role in its triggering or facilitation of its extension. This case highlights the importance of facilitation of bleeding complications by prior use of thrombolytic therapy not only peripherally but intracoronary too and the use of intravascular ultrasound for both diagnosis, followup, and percutaneous coronary intervention (PCI) guidance.

## 1. Introduction

The optimal treatment approach for coronary intramural hematomas has not been well defined, and discussion is limited to case reports [1–4]. In addition, the impact of prior thrombolytic therapy in the setting of coronary artery dissections with possible development and/or extension of an intramural hematoma is not well understood [1]. We present a case of acute anterior ST segment elevation myocardial infarction (STEMI), with initial good response to thrombolytic therapy and subsequent worsening due to iatrogenic periprocedural dissection of the LAD with development and extension of an intramural hematoma towards the LM and LCX arteries. Intramural hematoma was confirmed by intravascular ultrasound (IVUS) and successfully treated with multiple stents.

## 2. Case Report

A 42-year-old postmenopausal woman with no apparent cardiovascular risk factors had a history of non-ST segment elevation myocardial infarction with stenting of the proximal right coronary artery (RCA). Four months later, she presented to a remote hospital on an island in the North Sea with new onset retrosternal crushing typical chest pain associated with sweating. Her electrocardiogram (ECG) showed ST elevation and T-wave inversions in the anterior chest leads, and she was diagnosed as having an acute anterior STEMI. There were no capabilities to perform primary percutaneous coronary intervention (PCI) at that time or to transport her within the recommended time, and so the decision was taken to proceed with thrombolytic therapy. After receiving recombinant tissue plasminogen activator, she markedly improved with resolution of chest pain and ST segment normalization.

**Figure 1:**
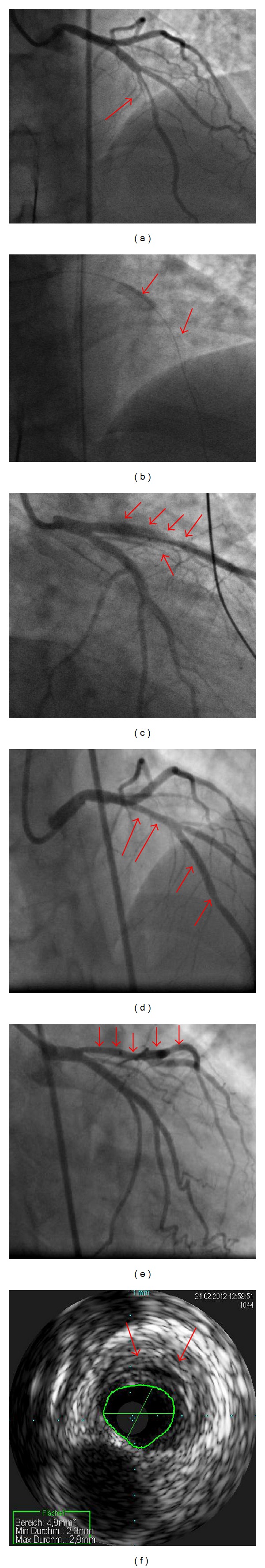
In (a), the mid LAD lesion is clearly seen before wiring. In (b) and (c), an iatrogenic dissection after stenting is clearly visible with proximal extension. The 2nd angiogram (d and e) shows narrowing of the LAD proximal to the placed stents. (f) shows IVUS confirmation of the diagnosis and estimation of minimum luminal area (4.8 mm²); so, the decision was made not to intervene based on IVUS result.

Next morning, angiography revealed a patent stent in the RCA and a critical mid LAD lesion just after the first diagonal bifurcation (D1). Ad hoc PCI was performed using 2 wires in LAD and D1 and direct stenting (without balloon predilatation) of the LAD with a drug-eluting stent. Thereafter, it appeared that the LAD lesion was not totally covered, and staining of contrast was visible proximally (possibly a dissection). Another distal stent was placed, while the dissection progressed proximally towards the LAD ostium. At this point, the procedure was aborted and a transfer to our hospital was arranged quickly (Figures 1(a)–1(c)). 

The patient had a reangiogram at the same day revealing a normal LM and LCX arteries, two patent nonoverlapping stents in the mid LAD with lumen narrowing proximally, and thrombolysis in myocardial infarction (TIMI) 3 flow. IVUS showed an intramural hematoma proximal to the stents with no dissection flaps and no significant lumen narrowing (Figures 1(d)–1(f)); so, we decided not to intervene as the patient became stable, and another control angiogram was scheduled after 3 days. Only Aspirin 100 mg and Clopidogrel 75 mg were given as antiplatelets with no other anticoagulants to try to avoid increase in the hematoma size. Full laboratory assessment was done with no abnormality apart from the elevated cardiac markers which were decreasing following the acute myocardial infarction. Full autoimmune profile was also performed due to suspicion of vasculitis but was later found to be normal. 

**Figure 2:**
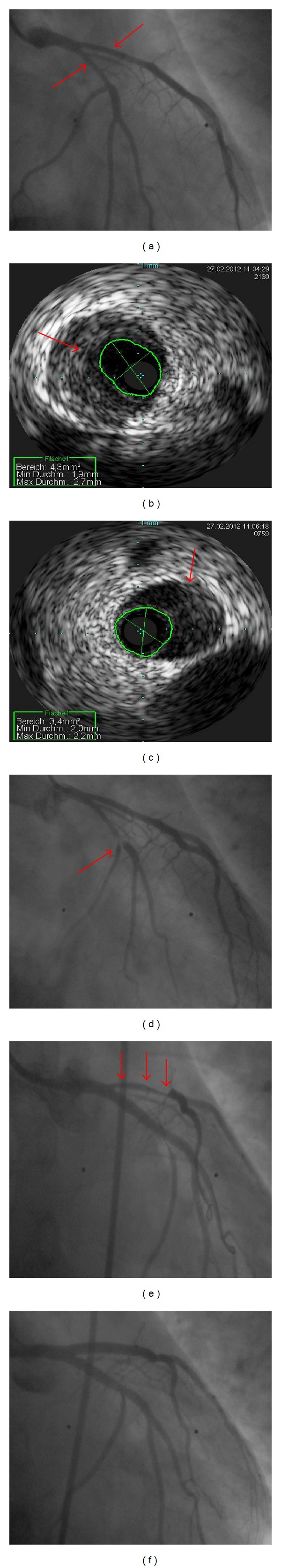
(a) clearly shows luminal narrowing in both proximal LAD and LCX in the control angiogram done 3 days later. (b) and (c) show IVUS confirmation of the diagnosis and estimation of minimum luminal area (4.3 mm² in LAD and 3.4 mm² in LCX). (d) shows proximal LCX tight lesion just after IVUS removal. (e) shows further luminal narrowing of the LAD due to milking of the hematoma after LCX-LM stenting. The final angiogram (f) shows the end result after LM-LAD and LM-LCX stenting (culotte technique).

Three days later, a control angiogram was done revealing clear increase in the size of the intramural hematoma in the LAD with a minimum lumen area of 4.3 mm^2^ (IVUS controlled) and clear progression of the hematoma towards the proximal LCX with a minimum lumen area of 3.4 mm^2^. After IVUS of the LCX, a critical lesion appeared at the site of IVUS with TIMI 1-2 flow distally not improving with intracoronary nitrates (assumed spasm) or balloon dilatation with a 1.5 and a 2.5 mm balloons (Figures 2(a)–2(d)).

An ad hoc heart team discussion supported a PCI approach. Thus, a 3.0 × 12 mm Xience Prime stent (Abbott Vascular, IL, USA) was placed in the proximal LCX, followed by another overlapping 3.5 × 18 mm Xience Prime stent to the LM trunk. The LAD ostium appeared compromised (shifting hematoma—Figure 2(e)); so, wires were exchanged, and balloon dilatation of the LM/LCX stent was done with a 2.0 mm balloon, and then, implantation of a 3.5 × 38 mm Xience Prime stent form the LM to the mid LAD (culotte technique) was performed. Rewiring of the LCX was done, and the procedure was finalized with kissing balloon dilatation with an excellent primary outcome (Figure 2(f)).

Echocardiography the next morning revealed upper normal left ventricular internal dimensions with impaired functions (ejection fraction of 35%) with anterolateral hypokinesia, akinetic apex, and the development of a small apical LV aneurysm with a small LV thrombus. Cardiac magnetic resonance imaging one week later confirmed the echocardiogram data in addition to myocardial edema at the infarction site with late enhancement indicating microvascular obstruction. The patient went through cardiac rehabilitation and is doing fine. She is scheduled for a control angiogram in 1 year.

## 3. Discussion

This is a case of iatrogenic periprocedural dissection of the LAD with development of an intramural hematoma and extension of this hematoma to the LM and LCX arteries, where prior recent thrombolytic therapy may have played a role in its triggering or facilitation of its extension. It was successfully detected by IVUS and treated with multiple stents. 

After PCI, intramural hematomas have been diagnosed in up to 6.7% of cases with intravascular ultrasound guidance [5]. Not all risk factors for intramural hematomas or coronary dissections have been identified, but there appears to be four groups of patients who present with intramural hematomas or coronary dissections: women in the peripartum period, atherosclerosis-associated coronary hematomas, percutaneous coronary intervention related, and idiopathic cases (including patients with connective tissue diseases, vasculitis, lupus erythematosus, extreme exercise, and also drug use) [3, 5, 6]. In the current case report, the dissection and intramural hematomas were PCI related. The European Society of Cardiology 2010 guidelines for myocardial revascularization recommends performing coronary angiography and PCI within 24 hours of giving thrombolytic therapy for STEMI patients (Class 1A) which is similar to the management here [7]. In this setting, not only peripheral vascular bleeding complications could occur but also coronary intramural hematomas may be facilitated especially following complicated PCIs.

A high awareness for intramural hematomas or dissections is required when performing PCI as a hematoma may simply appear as diffuse coronary luminal narrowing. A new stent-edge lesion or milking of a *de novo *coronary lesion distally, proximally or into an adjacent branch or vessel has been described previously [8]. The use of IVUS can identify intimal tears and provide important information about the length of the dissection, vessel size, the development and extension of an intramural hematoma, and the adequate compression of intramural hematoma after PCI. Furthermore, the use of IVUS can guide PCI and can minimize the incidence of PCI-related complications [9]. Vessels facilitating the extension of a hematoma are typically relatively healthy, thereby allowing propagation of blood within the media without being impeded by calcific or fibrotic plaques [5], which was clearly apparent in this case. We assume that the major contributors for this complication were the recent thrombolytic therapy, the relatively healthy vessel wall, and the fact that the dissection was not sealed proximally.

Although there is no clear consensus on management of multivessel intramural hematomas, the key principal of re-establishing coronary flow in the setting of ongoing ischemia holds true as in any acute coronary syndrome (ACS). If vital conditions are stable and ischemia is not ongoing, intramural coronary hematoma may be treated medically similar to medical treatment of ACS. Thrombolytic therapy in patients with coronary dissections and intramural hematomas is not recommended due to the possibility of extension resulting in poor prognosis [10].

## 4. Conclusion

Early PCI postthrombolytic therapy can facilitate vascular bleeding complications not only peripherally but also intramural coronary hematomas may develop and extend, which may lead to a poor clinical outcome.
